# Chronic d-serine supplementation impairs insulin secretion

**DOI:** 10.1016/j.molmet.2018.07.002

**Published:** 2018-07-25

**Authors:** Lisa Suwandhi, Simone Hausmann, Alexander Braun, Tim Gruber, Silke S. Heinzmann, Eric J.C. Gálvez, Achim Buck, Beata Legutko, Andreas Israel, Annette Feuchtinger, Elizabeth Haythorne, Harald Staiger, Martin Heni, Hans-Ulrich Häring, Philippe Schmitt-Kopplin, Axel Walch, Cristina García Cáceres, Matthias H. Tschöp, Guy A. Rutter, Till Strowig, Martin Elsner, Siegfried Ussar

**Affiliations:** 1 RG Adipocytes & Metabolism, Institute for Diabetes & Obesity, Helmholtz Center Munich, 85748 Garching, Germany; 2 German Center for Diabetes Research (DZD), 85764 Neuherberg, Germany; 3 Institute of Groundwater Ecology, Helmholtz Center Munich, 85764 Neuherberg, Germany; 4 Institute for Diabetes & Obesity, Helmholtz Center Munich, 85748 Garching, Germany; 5 Research Unit Analytical BioGeoChemistry, Department of Environmental Sciences, Helmholtz Center Munich, 85764 Neuherberg, Germany; 6 Research Group Microbial Immune Regulation, Helmholtz Centre for Infection Research, Braunschweig, Germany; 7 Research Unit Analytical Pathology, Helmholtz Center Munich, 85764 Neuherberg, Germany; 8 Section of Cell Biology and Functional Genomics, Department of Medicine, Imperial College London, London, UK; 9 Institute of Pharmaceutical Sciences, Department of Pharmacy and Biochemistry, Eberhard Karls University Tübingen, 72076 Tübingen, Germany; 10 Institute for Diabetes Research and Metabolic Diseases of the Helmholtz Center Munich at the Eberhard Karls University Tübingen, 72076 Tübingen, Germany; 11 Department of Internal Medicine, Division of Endocrinology, Diabetology, Angiology, Nephrology, and Clinical Chemistry, University Hospital Tübingen, 72076 Tübingen, Germany; 12 Analytical Chemistry and Water Chemistry, Technical University of Munich, 81377 Munich, Germany; 13 Division of Metabolic Diseases, Department of Medicine, Technische Universität München, Germany

**Keywords:** d-serine, Diabetes, Obesity, Insulin secretion

## Abstract

**Objective:**

The metabolic role of d-serine, a non-proteinogenic NMDA receptor co-agonist, is poorly understood. Conversely, inhibition of pancreatic NMDA receptors as well as loss of the d-serine producing enzyme serine racemase have been shown to modulate insulin secretion. Thus, we aim to study the impact of chronic and acute d-serine supplementation on insulin secretion and other parameters of glucose homeostasis.

**Methods:**

We apply MALDI FT-ICR mass spectrometry imaging, NMR based metabolomics, 16s rRNA gene sequencing of gut microbiota in combination with a detailed physiological characterization to unravel the metabolic action of d-serine in mice acutely and chronically treated with 1% d-serine in drinking water in combination with either chow or high fat diet feeding. Moreover, we identify SNPs in SRR, the enzyme converting L-to d-serine and two subunits of the NMDA receptor to associate with insulin secretion in humans, based on the analysis of 2760 non-diabetic Caucasian individuals.

**Results:**

We show that chronic elevation of d-serine results in reduced high fat diet intake. In addition, d-serine leads to diet-independent hyperglycemia due to blunted insulin secretion from pancreatic beta cells. Inhibition of alpha 2-adrenergic receptors rapidly restores glycemia and glucose tolerance in d-serine supplemented mice. Moreover, we show that single nucleotide polymorphisms (SNPs) in SRR as well as in individual NMDAR subunits are associated with insulin secretion in humans.

**Conclusion:**

Thus, we identify a novel role of d-serine in regulating systemic glucose metabolism through modulating insulin secretion.

## Highlights

• d-serine suppresses HFD induced weight gain.
• d-serine supplementation impairs insulin secretion.
• d-serine effects on insulin secretion can be reverted by a2 adrenergic antagonists.

## 1 Introduction

Type 2 diabetes (T2D) is a multifactorial metabolic disease, which, as shown by a large number of studies including genome-wide association studies (GWAS) [1], [2], [3] as well as numerous large scale metabolite screens [4], [5] depends on complex gene and environment interactions. To date, single nucleotide polymorphisms (SNPs) in more than 100 genes have been described to contribute to the development of T2D [3]. Moreover, the list of host or environment derived metabolites contributing to the development of T2D is continuously growing [6], [7], [8]. Importantly, many of the identified SNPs and metabolites associate with alterations in beta cell function and insulin secretion [9], [10], [11]. However, some of these proteins and metabolites are also associated with the development of other diseases not directly linked to beta cell function [12]. One of those genes is serine racemase (SRR) [13], which catalyzes the conversion from l-serine to d-serine, an important co-agonist of N-methyl-d-aspartate (NMDA) receptors. SRR function was recently suggested to regulate insulin secretion from pancreatic beta cells [3], [14], and chronic as well as acute supplementation of the SRR product d-serine reduced food intake and weight gain upon high fat diet (HFD) feeding in mice [15], [16]. However, dysfunction of SRR, the d-serine degrading d-amino acid oxidase (DAO), and the main d-serine transporter alanine-serine-cysteine transporter 1 (Asc-1) are implicated in the development of schizophrenia, Alzheimer's disease, and depression [17], [18], [19], [20], [21]. However, a more detailed knowledge on the metabolic actions of d-serine could contribute to a better understanding of the strong linkage between obesity, T2D, and schizophrenia [22].

Here, we report positive and negative consequences of long-term treatment with d-serine. We show that d-serine suppresses high fat diet intake resulting in reduced weight gain. Moreover, we demonstrate that highly dosed d-serine induces hyperglycemia, and strongly impairs glucose tolerance due to impaired insulin secretion from pancreatic beta cells. This depends on increased sympathetic activity in the islets of Langerhans rather than direct action on beta cells and can be completely reversed by alpha 2-adrenergic receptor antagonists. Furthermore, we identify novel SNPs in SRR as well as several NMDAR subunits to associate with insulin secretion in humans. Thus, alterations in d-serine levels could contribute to the development of T2D through the modulation of insulin secretion, especially in genetically predisposed subjects.

## 2 Material and methods

### 2.1 Mouse models

C57BL/6N-Rj male mice were purchased from Janvier at age 3 weeks or 7 weeks. Germfree mice were bred at the HZI in Braunschweig. The mice were kept in groups of at least 4 under a 12 h light: 12 h dark cycle and an ambient temperature of 22 ± 2 °C. Mice were fed a standard laboratory chow diet (Altromin 1314) or a 58% high fat diet (Research Diets D12331) *ad libitum*. Animals received either normal water or water supplemented with 1% d-serine (Sigma) or/and 0.3% Dextromethorphan hydrobromide monohydrate (DXM; Sigma) *ad libitum*. To study acute oral uptake of d-serine, eight weeks old male C57Bl/6 mice were gavaged with 100 mg d-serine/kg body weight. For the food preference test, mice were starved for 16 h and afterwards refed with CD and HFD. After 3 h, the weight of the consumed food was determined. Body composition was analyzed with a non-invasive magnetic-resonance whole-body composition analyzer (EchoMRI). Energy expenditure, food and water consumption, locomotor activity, and respiratory exchange quotient (RER) was measured with an indirect calometric system (TSE PhenoMaster). Fecal caloric content was measured from dried fecal pellets using a 6300 Oxygen Bomb Calorimeter (Parr Instrument Technology). Animal experiments were conducted in accordance with the German animal welfare law and performed with permission and in accordance with all relevant guidelines and regulations of the district government of Upper Bavaria (Bavaria, Germany), protocol number 55.2-1-54-2532-52-2016.

### 2.2 Glucose, insulin, pyruvate tolerance tests and assessment of glucose stimulated insulin secretion

Glucose tolerance tests (GTTs) and insulin tolerance tests (ITTs) were carried out in mice fasted for 4 h. 2 g/kg glucose (20% Glucose solution; Braun) or 0.75 U insulin/kg BW (Actrapid® PenFill® Novo Nordisk) were injected intraperitoneally (i.p.) and glucose concentrations measured in blood collected from the tail before and 15, 30, 60, and 120 min after the injection using a FreeStyle Freedom Lite glucometer (Abbott). Glucose stimulated insulin secretion (GSIS) was assessed in mice fasted for 4 h prior to the i.p. injection of 5 mg/kg BRL44408 (abcam) and 4 g/kg glucose. Blood was collected from the tail vein before and 5, 10, 15, and 30 min after the injection. At the same time points, glucose concentrations were measured. Serum was prepared by centrifugation (5 min 10.000 × g 4 °C) and serum insulin concentrations were determined using the mouse ultrasensitive insulin ELISA kit (Alpco). For the pyruvate tolerance test (PTT), mice were fasted for 16 h and 5 mg/kg sodium pyruvate (Sigma–Aldrich) were injected i.p. and glucose concentrations were measured before and 15, 30, 60, and 120 min after the injection.

### 2.3 d-serine measurement

d-serine, l-serine, sodium tetraborate, Marfey's reagent (N_α_-(2,4-dinitro-5-fluorophenyl)-l-alaninamide), hydrochloric acid, and acetone were purchased from Sigma–Aldrich. Acetonitrile was purchased from Thermo Scientific. LC-MS grade water with 0.1% acetic acid was purchased from Fluka.

Tissues were homogenized in 1 ml 100% methanol using a Polytron and dried at 60 °C under a stream of N_2_, and the dried residues were reconstituted in 50 μl milliQ water. Separation of the serine enantiomers was performed with chemical derivatization using Marfey's reagent, generating dinitrophenyl-5-l-alanine-d/l-serine diastereomers (DNPA-D/l-serine), which can be separated by reverse-phase chromatography [23]. To this end, 100 μl methanol, 50 μl 0.125 M sodium tetraborate buffer, and 50 μl 1% Marfey's reagent in acetone were added to the reconstituted sample and heated to 50 °C for 60 min. Derivatization was then stopped by addition of 20 μl 2N HCl. For standard curve calibration, D- and l-serine standards were prepared at 166, 55, 18, 6, 2, 0.7, 0.2, 0.07, and 0.02 mg/l by serial dilution of a 500 mg/l stock solution and derivatized as described above.

Chromatographic separation of DNPA-D- and DNPA-l-Serine was accomplished on an Agilent HP 1200 HPLC system using a Synergi Hydro-RP (C18) 150 mm × 2.1 mm I.D., 4 μm 80°A particles column (Phenomenex) at 25 °C. Mobile phase A was LC-MS grade water with 0.1% acetic acid and mobile phase B was acetonitrile, which was delivered according to the following gradient profile (min/% mobile phase B): 0.0/5, 1.9/5, 12/20, 20/20, 24/90, 30/90. The flow rate was 0.2 ml/min and the injection volume was 10 μl.

The HPLC system was coupled to a Q-Trap MS/MS system (Applied Biosystems). Ionization was accomplished by electrospray ionization in the negative ion mode. Mass spectrometry was carried out in the multiple reaction monitoring mode. To determine the MS/MS fragment pattern with the highest intensity, a single analyte standard containing DNPA-D/l-serine was dissolved in a mixture of water and methanol 50:50 (v/v) at a concentration of 1 mg/l. The standard was infused at a flow rate of 200 μl/min for tuning the compound-dependent MS parameters. The infusion experiment was performed using a syringe pump directly connected to the interface. Optimal detection was provided by scanning for the mass pair 356/356. Declustering potential, collision energy, collision cell exit potential, and entrance potential were optimized to −45, −12, −3, and −6 V. The optimized values for the parameters ion spray voltage, nebulizer gas, auxiliary gas, curtain gas, collision gas and auxiliary gas temperatures were −4500, 40, 35, 20, 3, and 400 °C, respectively. The curtain, collision, turbo, and nebulizer gas was nitrogen generated from pressurized air in a nitrogen generator. To obtain adequate selectivity and sensitivity, the mass spectrometer was set to unit resolution, and the dwell time was 150 ms.

Calibration curves were obtained by plotting the peak area of D/l-serine against their concentration. Peak integration was automatically accomplished via Analyst software (version 1.4.2, AB/MDS Sciex). A least squares regression analysis was used to obtain a linear equation over the range of the calibration. The assay finally produced a standard curve that was linear over a range of 0.02–166 mg/l. Samples were measured in triplicates.

### 2.4 Gut microbiota analysis

Mouse fecal pellets were collected and preserved at −20 °C until processing. DNA was isolated using an established protocol [24]. Briefly, the samples were suspended in 500 μl of extraction buffer (200 mM Tris, 20 mM EDTA, 200 mM NaCl, pH 8.0), 200 μl of 20% SDS, 500 μl of phenol:chloroform:isoamyl alcohol (24:24:1), and 100 μl of zirconia/silica beads (0.1 mm diameter). Bacterial cell disruption was done with a bead beater (BioSpec) twice for 2 min. After isopropanol precipitation, crude DNA extracts were resuspended in TE Buffer with 100 μg/ml RNase I and furthermore column purified (BioBasic).

PCR amplification targeting the V4 region (F515/R806) of the 16S rRNA gene was performed according to previously described protocols [25]. In short, 25 ng of DNA were used per PCR reaction (30 μl), the PCR conditions consisted of initial denaturation for 30 s at 98 °C, followed by 25 cycles (10 s at 98 °C, 20 s at 55 °C, and 20 s at 72 °C). Each sample was amplified in triplicates and subsequently pooled. After normalization, PCR amplicons were sequenced on an Illumina MiSeq platform (PE250).

Obtained reads were assembled, quality controlled, and clustered using Usearch8.1 software package (http://www.drive5.com/usearch/). Briefly, reads were merged using -fastq_mergepairs –with fastq_maxdiffs 30 and quality filtering was done with fastq_filter (-fastq_maxee 1), minimum read length 200 bp. The OTU clusters and representative sequences were determined using the UPARSE algorithm [26], followed by taxonomy assignment using the Silva database v128 [27] and the RDP Classifier [28] with a bootstrap confidence cutoff of 80%. The OTU absolute abundance table and mapping file were used for statistical analyses and data visualization in the R statistical programming environment (R Core Team 2016) package phyloseq [29].

### 2.5 Serum and liver metabolites

Serum insulin levels were determined using the Mouse ultrasensitive insulin ELISA kit (Alpco). C-peptide was measured with usage of the C-peptide (mouse) ELISA kit (Biocat). Liver triglyceride content was measured with the Triglyceride Quantification Colorimetric/Fluorometric Kit (BioVision), and liver glycogen content was quantified with the starch assay (r-biopharm, Darmstadt, Germany).

For determination of the liver metabolomics, metabolites from 50 mg of liver tissue were extracted with an aqueous extraction procedure. 1 ml of H_2_O was added to the sample with ceramic beads (NucleoSpin, Macherey–Nagel, Dueren, Germany) and homogenized for 3 min at 30 1/s in a TissueLyser II (Qiagen, Hilden, Germany). Samples were centrifuged (5 min, 13000 × g), and the supernatant was taken as aqueous extract. 100 μl of the extract was mixed with 50 μl NMR buffer (90% D2O, 500 mM PO4 buffer with 0.1% trimethylsilyl-tetradeuteropropionic acid [TSP], pH 7.4), and analyzed immediately by NMR spectroscopy.

All samples were transferred to 3 mm outer diameter NMR Bruker Match tubes (Hilgenberg GmbH) and analyzed in a randomized order. NMR spectra were acquired on a Bruker 800 MHz spectrometer (Bruker Biospin) operating at 800.35 MHz with a quadruple inverse (QCI) cryogenic probe. For an overview of all molecules present in the sample, a standard one-dimensional (1D) pulse sequence [recycle delay (RD), 90°, t1, 90°, mixing time (tm), 90°, acquire FID] was acquired, with water suppression irradiation during an RD of 2 s, the tm set on 200 ms, and a 90° pulse set to 9 μs, which collected 512 scans into 64 K data points with a spectral width of 12 ppm. A representative sample of each liver section underwent 2D NMR analysis for detailed metabolite analysis and metabolite identification. For the 2D 1H−13C HSQC spectra, phase-sensitive ge-2D HSQC using PEP and adiabatic pulses for inversion and refocusing with gradients were used. For each 2D spectrum, 4096 × 1024 data points were collected using 64 scans per increment, an acquisition time of 0.25 s, and 16 dummy scans. The spectral widths were set to 12 and 230 ppm in the proton and carbon dimensions, respectively. For the 2D 1H−1H TOCSY spectra, phase sensitive sensitivity-improved 2D TOCSY with WATERGATE (3-9-19) and DIPSI-2 were acquired. For each 2D spectrum, 19,228 × 1024 data points were collected using 16 scans per increment, an acquisition time of 1 s, and 16 dummy scans. The spectral widths were set to 12 and 12 ppm in the F2 and F1 dimensions, respectively. Processing of the spectra was carried out in a TopSpin 3.2 (Bruker BioSpin). FIDs were multiplied by an exponentially decaying function corresponding to a line broadening of 0.3 Hz before the Fourier transformation. All spectra were manually phased, baseline corrected, and calibrated to TSP (δ 0.00). Data were imported into Matlab (Mathworks) and further processed (i.e., the water region was removed). Quantification of selected metabolites was done by integration of their area under the curve (AUC) and referencing to the AUC of TSP (in mmol) and the liver wet weight (in kg).

### 2.6 Histology and imaging

Tissues were fixed in 4% PFA/PBS overnight, paraffin embedded and sectioned 2–5 μm. Sections were stained with hematoxylin and eosin or Sirius Red [30] to assess fibrosis. Immunohistochemistry for insulin (1:50; Santa Cruz Biotechnology sc-9168) was performed using a VECTASTAIN ABC HRP Kit following the manufacturer's instructions with a Mayer's Hemalum counterstain. Adipocyte size was calculated from measuring all adipocytes per slide from a H&E staining and islet size based on the insulin staining using a Mirax Desk digital slide scanner (Carl Zeiss MicroImaging) and the Definiens Developer XD2 (Definiens AG) software. To assess α-, β-, and δ-cell distribution, pancreas was embedded in OCT (Sakura), and 5 μm sections were fixed 10 min in 4% PFA, washed 3 × with PBS, and permeabilized (0,1 M Glycine, 0,2% TritonX-100 in PBS) for 30 min. After blocking for 1 h in donkey serum, primary antibodies were added over night at 4 °C (rabbit α-Insulin (Cell Signaling; 1:300), goat α-Nkx6.1 (R&D Systems; 1:300), guinea pig α-Glucagon (TaKaRa; 1:2500) in donkey block). Secondary antibodies (α-rabbit Alexa 488 (Invitrogen; 1:800), α-goat Alexa 555 (Invitrogen; 1:800), α-guinea pig Alexa 647 (Invitrogen; 1:188), DAPI (Sigma Aldrich; 1:5000) in donkey block) were added for 1 h and stainings mounted in Fluorescent mounting medium (DAKO, S3023). Images were acquired using a Zeiss scope A1 and a Leica SP5 confocal microscope.

For cFos stainings, animals were fasted for 6 h, sacrificed with CO_2_, and transcardially perfused with saline (0.9% NaCl) followed by 4% cold paraformaldehyde (PFA) in phosphate buffered saline (pH 7.4). Brains were removed and post-fixed in 4% PFA at 4 °C before being equilibrated for 48 h with 30% sucrose in Tris-buffered saline (pH 7.2). Brains were coronally cut into 40 μm sections at a cryostat, washed in TBS and incubated overnight with primary antibody anti-cFOS (rabbit polyclonal, 1:500, Santa Cruz Biotechnology, Inc, TX, US) diluted in SUMI (gelatin 0.25% and TritonX-100 0.5% in TBS) at 4 °C. After serial washes in TBS, sections were incubated with secondary antibody (alexa fluor 647 donkey-anti-rabbit (1:1000 in SUMI, Molecular Probes, Life Technologies GmbH, Darmstadt, Germany), washed with TBS, mounted on gelatin-pre-coated glass slides and kept in the dark and 4 °C for drying. Dried sections were mounted with a coverslip using Mowiol-based mounting solution containing the anti-fading agent DABCO (*elvanol*). Images were captured as z-stacks with 2 μm steps at 10 × magnification by a Leica TCS SP5 II confocal microscope (Leica Microsystems, Wetzlar, GER). Quantification of c-Fos immunoreactive (cFOS+) cells was performed using ImageJ software. Maximum projections of confocal scans were automatically thresholded, and the number of cFos + cells per each hypothalamic nucleus according with the mouse brain atlas (PVN, ARC, DMN, VMN and LH) was determined. All morphometric analyses were performed without previous knowledge of the experimental group and nuclear staining was counted in blinded fashion.

### 2.7 Matrix-assisted laser desorption/ionization Fourier-transform ion cyclotron resonance mass spectrometry imaging (MALDI FT-ICR MSI)

Organs were dissected and immediately frozen in liquid nitrogen. To gather information about distribution of ^13^C-labeled d-serine, frozen tissue samples were cryosectioned into 12 μm thick slices and thaw-mounted onto pre-cooled conductive indium tin oxide-coated glass slides (Bruker Daltonics) pre-coated with a 1:1 mixture of poly-l-lysine and 0.1% Nonidet P-40 (Sigma–Aldrich). Tissue sections were coated with 9-aminoacridine hydrochloride monohydrate matrix (Sigma–Aldrich) at 10 mg/ml in methanol/H_2_O (7:3, v/v) using a SunCollect sprayer (Sunchrom). Matrix solution was deposited in eight layers onto the tissue section with the flow rates at 10, 20, 30, and 40 μl/min. MALDI MSI was performed in negative ion mode on a Bruker Solarix 7T FT-ICR MS (Bruker Daltonics). Accurate mass measurements were acquired in negative mode using continuous accumulation of selected ions with a mass window of *m/z* 50–150, a 2 M data point transient (0.5243 s duration) providing a mass resolving power of approximately 300,000 at *m/z* 100. The Smartbeam-II Nd:YAG (355 nm) laser operated at a frequency of 1000 Hz using 200 laser shots per spot and a lateral resolution of 60 μm. After MALDI-MSI completion, the matrix was removed from slides with 70% ethanol, and tissue sections were stained using hematoxylin and eosin. The resulting data were analyzed using FlexImaging 4.0 (Bruker Daltonics) with root mean square normalization of the ion images.

### 2.8 Isolation of mouse pancreatic islets

Mice were sacrificed by cervical dislocation, and the bile duct was clamped. 4 ml of cold collagenase solution (1 mg/ml Collagenase P in G-solution) was injected into the duct. The pancreas was removed and incubated in 6 ml collagenase solution for 12 min at 37 °C. 10 ml of cold G-solution (1% BSA, 1% PenStrep in HBSS) was added to the digested pancreas and centrifuged 2 min 290 × g. Pellet was washed with 10 ml G-solution and after another centrifugation resuspended in 11 ml 15% Optiprep (60% Optiprep (Sigma), 3,75 mM Hepes, 10% RPMI in HBSS + PenStrep). Gradient was pipetted (5 ml 15% Optiprep, 11 ml cell suspension, 12 ml G-solution) and centrifuged 15 min 226 × g without break. Islet containing phase was filtered (70 μm strainer) and islets were picked and cultured in RPMI over-night.

### 2.9 Insulin secretion from pancreatic islets

10 islets per well were incubated in a 96 v-bottom well plate at 37 °C for 1 h in 100 μl KRB (120 mM NaCl, 4.8 mM KCl, 2.5 mM CaCl_2_ × 2H_2_O, 1.2 mM MgCl_2_, 5 mM Hepes, 24 mM NaHCO3, 0.1% BSA) containing 2 mM glucose and combinations with 400 μM d-serine and 10 μM dextrorphan tartrate (DXO). After 1 h, the supernatant was discarded and islets were incubated with the same conditions for 1 h. Supernatant was collected (low glucose), and islets were incubated for 1 h at 37 °C with KRB containing 16.7 mM glucose and supplements as above. Supernatant was collected (high glucose) and stored at −20 °C. Remaining islets were lysed in 500 μl Acid-Ethanol (70% Ethanol + 1.5% HCl) using the sonicator and incubated at 4 °C over-night. Lysed cells were centrifuged (7000 rpm, 4 °C, 10 min), and the supernatant transferred into a new tube and stored at −20 °C. Insulin concentrations were determined using the Mouse insulin ELISA (AppliChem), and secreted insulin was normalized to total insulin content.

### 2.10 Islet membrane potential measurements

Pancreatic islets were dissociated into single β-cells and plated onto glass coverslips, as previously described [31]. Electrophysiological recordings were performed in perforated patch-clamp configuration using an EPC9 patch-clamp amplifier controlled by Pulse acquisition software (Heka Elektronik). β-cells were identified morphologically and by depolarization of the membrane potential in response to 16.7 mM glucose. β-cells were constantly perfused at 32–33 °C with normal saline solution (mM): 135 NaCl, 5 KCl, 1 MgCl_2_, 1 CaCl_2_, 10 HEPES, and 16.7 glucose (pH 7.4). Recording electrodes had resistances of 8–10 MΩ and were filled with a solution comprised of (mM): 140 KCl, 5 MgCl_2_, 3.8 CaCl_2_, 10 HEPES, 10 EGTA (pH 7.2), and 20–25 μg/ml amphotericin B (Sigma–Aldrich).

### 2.11 Human subjects and genotype–phenotype association analysis

The ongoing Tübingen Family (TÜF) study for type-2 diabetes currently includes more than 3,000 non-diabetic Caucasian individuals at increased risk for type-2 diabetes (subjects with family history of diabetes, BMI ≥ 27, impaired fasting glycemia, or previous gestational diabetes). The participants were characterized by anthropometrics, 5-point oral glucose tolerance tests (OGTTs), and clinical chemistry [32] and genome-wide genotyped with Illumina's Infinium® Global Screening Array-24 v1.0 BeadChip, which depicts 700,078 single nucleotide polymorphisms (SNPs). From the TÜF study, we selected 2,670 subjects with complete phenotypic datasets (gender, age, BMI, insulin sensitivity, insulin secretion) as study population for analysis of the human genes *SRR*, *DAO*, *SLC7A10*, *SHMT1*, *GRIN1*, *GRIN2A*, *GRIN2B*, *GRIN2D*, *GRIN3A*, and *GRIN3B* (for *GRIN2C*, no SNPs were depicted on the array). From this set of genes, 137 non-linked (r^2^ < 0.8), bi-allelic SNPs in Hardy–Weinberg equilibrium (p ≥ 0.05, χ^2^-test) with minor allele frequencies ≥0.05 and genotyping success rates ≥75% were analyzed. Genotype-phenotype analyses were performed by multiple linear regression analysis (least squares method) with insulin secretion (area under the curve [AUC] C-peptide _0′–30′ OGTT_/AUC glucose _0′–30′ OGTT_) as dependent variable, the SNP genotype in the additive inheritance model as independent variable, and gender, age, BMI, and insulin sensitivity as confounding covariates. According to Bonferroni correction for the number of SNPs tested in parallel, p-values <0.000375 were considered significant. Associations were indicated as nominal if p-values were ≥0.000375 and <0.05. The study followed the principles laid down in the Declaration of Helsinki and was approved by the Ethics Committee of the University of Tübingen. Informed written consent was obtained from all participants.

### 2.12 Statistics

Data are presented as mean ± standard error of the mean (SEM) unless stated differently in the figure legend. Statistical significance was determined by unpaired Student's t-test or, for multiple comparisons, using One- or Two-Way ANOVA, followed by Tukey's Multiple Comparison's Test, or as stated in the respective figure legend. Differences reached statistical significance with p < 0.05. For NMR spectroscopy, multivariate statistics, i.e. orthogonal partial least-squares discriminant analysis (OPLS-DA), were carried out in order to discriminate liver samples from mice under different serine supplementation. Here, OPLS loading plots show metabolites across the whole non-targeted metabolite analysis that differ between the treatment groups. These metabolites were then quantified and univariate statistical analysis carried out.

## 3 Results

### 3.1 d-serine supplementation ameliorates diet induced obesity and preference to HFD

**Figure 1:**
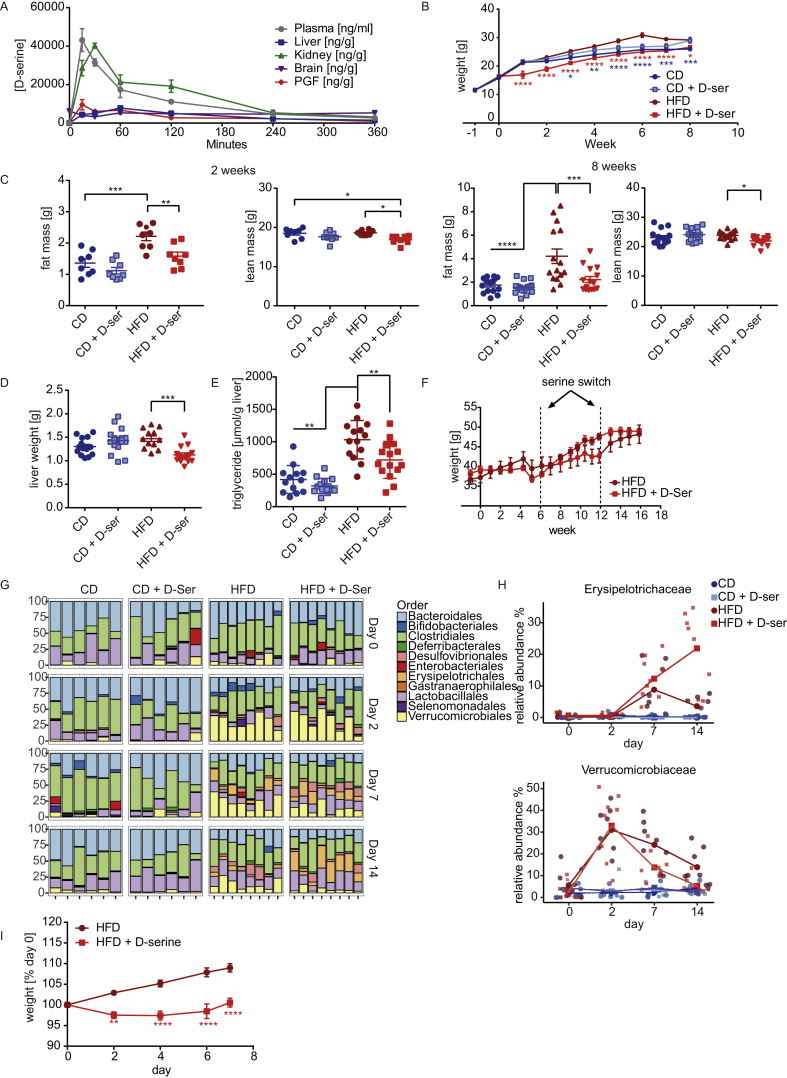
**d****-serine regulates food preference and HFD induced weight gain**. (**A**) 8 week old C57Bl/6 mice were gavaged with 100 mg d-serine/kg body weight or saline as controls. Mice gavaged with saline were used as controls and shown as time 0. Groups of four mice gavaged with d-serine were sacrificed after 15, 30, 60, 120, 240, and 360 min after administration and d-serine concentrations were measured by mass spectrometry. (**B**) Body weight curves of 4 week old C57Bl/6 mice fed a CD or HFD, and drinking water supplemented with or without 10 g/l d-serine throughout 8 weeks (n = 7–8). (**C**) Body composition after 2 and 8 weeks of serine supplementation (n = 7–16). (**D**) Liver weights after 8 weeks of treatment (n = 15–16). (**E**) Liver triglyceride content after 8 weeks of d-serine supplementation (n = 15–16). (**F**) Weight gain during 15 weeks of serine supplementation after 7 weeks on HFD (n = 4). After 6 and 12 weeks, the water bottles were switched. (**G**) Relative microbiota abundance at order level. The bar plot is displayed by treatment and day of intervention (n = 6–8). (**H**) Relative abundance dynamics of bacterial biomarkers Erysipelotrichaceae and Verrucomicrobiaceae (Akkermansia). (**I**) Weight gain in germfree C57Bl/6 mice fed with HFD with or without 1% d-serine supplementation (n = 5). Data are shown as mean ± SEM. Statistics were calculated either by one-way or two-way ANOVA with Tukey's or Sidak's (for germfree mice) multiple comparison post-hoc test (****P < 0.0001, ***P < 0.001, **P < 0.01, *P < 0.05).

Following oral gavage, d-serine rapidly appeared in plasma and perigonadal fat (PGF) with peak concentrations after 15 min followed by appearance in kidneys at 30 min and liver after 60 min (Figure 1A). In contrast, d-serine was detectable in whole brain homogenates at baseline, without increase upon exogenous administration. Tissue concentrations of d-serine returned to baseline within six hours. To investigate the long-term metabolic effects of d-serine supplementation, four week old male C57Bl/6 mice were either fed a regular chow diet (CD) or HFD (58% calories from fat) with or without (control) supplementation of 1% d-serine in drinking water for eight weeks. d-serine supplementation did not result in statistically significant alterations in weight gain in CD fed mice (Figure 1B). In contrast, mice fed HFD + d-serine showed strongly reduced weight gain during the first week of supplementation with paralleled weight gain to HFD fed mice, but no catch up thereafter (Figure 1B). After 8 weeks on diet, HFD + d-serine fed mice had the same weight as CD control animals. Assessment of body composition after two and eight weeks of treatment revealed that the reduced body weight of HFD + d-serine fed mice was primarily due to decreased fat mass (Figure 1C), which was also reflected by reduced subcutaneous (SCF), perigonadal (PGF), and brown (BAT) adipose tissue mass (Figure S1A). HFD + d-serine fed mice also showed a small but significant decrease in lean mass compared to controls (Figure 1C), albeit body length was the same (Figure S1B). In line with reduced adipose tissue mass, liver weight (Figure 1D), and liver triglyceride content (Figure 1E) were significantly reduced in HFD + d-serine fed compared to HFD control mice after eight weeks on diet. Water intake was not altered at either time point or diet group (Figure S1C–D). d-serine supplementation following the establishment of diet induced obesity also blunted weight gain (Figure 1F) and food intake (Figure S1E). Moreover, switching d-serine supplementation between control and d-serine supplemented mice rapidly reversed weight gain and food intake repeatedly (Figure 1F and Figure S1E).

d-serine can be produced and utilized by gut microbiota, potentially affecting overall community structure and activity [33], which could modulate weight gain [34]. Thus, we analyzed fecal gut microbiota at days 0, 2, 7, and 14 in mice fed either CD or HFD with or without d-serine. HFD feeding resulted in rapid reconfiguration of gut microbiota within two days, especially in the abundance of Verrucomicrobiales (Figure 1G and Figure S1F–I). However, d-serine did not have a significant effect on overall variability as shown by permutational multivariate analysis of variance, or α-diversity (Figure S1G–H). When comparing d-serine supplemented to control animals on HFD, we only observed effects of d-serine on gut microbiota at day 14, showing an increase in members of the family Erysipelotrichaceae and a reduction in members of the family Verrucomicrobiaceae (Figure 1G–H and Figure S1G). To test if changes in specific bacterial species not captured by 16S rRNA analysis or their activity contribute to the altered weight gain, germfree C57Bl/6 mice were fed a HFD with or without d-serine supplementation. Similar to what was observed in conventionally raised mice, d-serine supplementation blunted weight gain in germfree mice (Figure 1I) with no differences in fecal caloric content (Figure S1J). Hence, d-serine is rapidly taken up into the circulation and various peripheral organs and regulates food preference and HFD induced body weight gain, but gut microbiota do not seem to directly contribute to the reduced HFD induced weight gain by d-serine.

### 3.2 d-serine administration induces diet independent glucose intolerance but not insulin resistance

**Figure 2:**
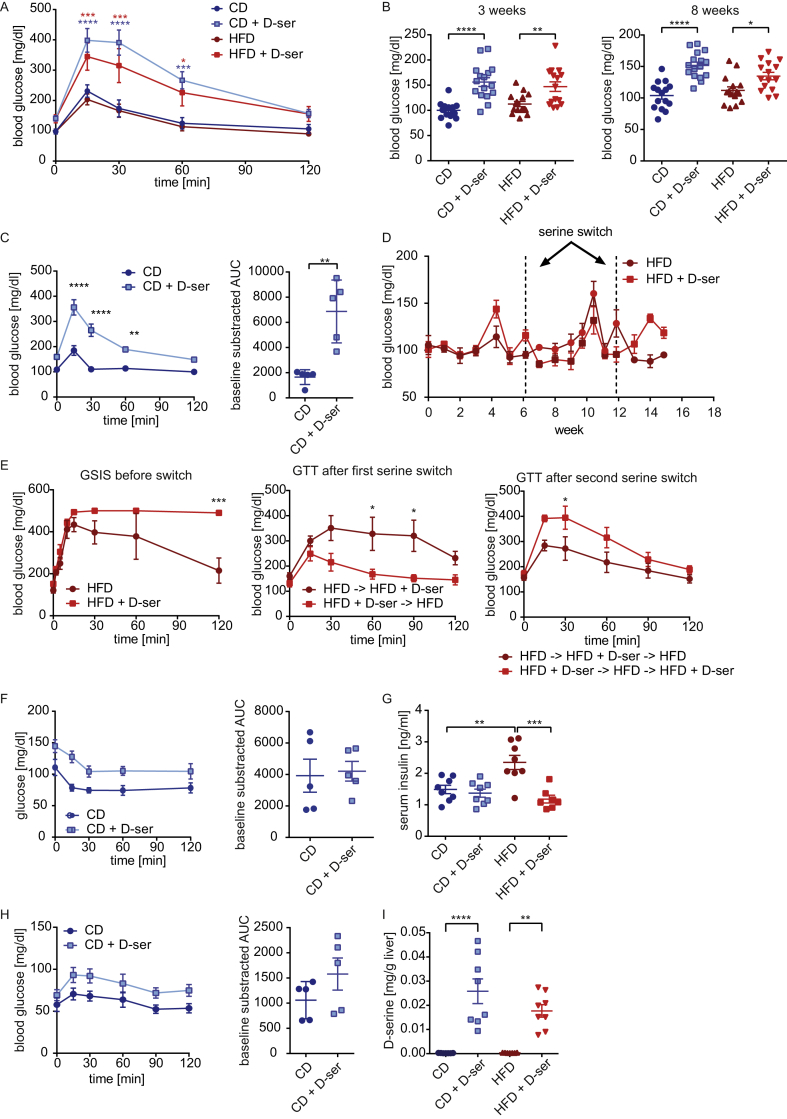
**d****-serine supplementation impairs glucose but not insulin tolerance**. (**A**) ipGTT after 6 weeks of d-serine supplementation (n = 7–8). (**B**) Blood glucose levels after three (random fed) and eight (4 h fasted) weeks of d-serine supplementation (n = 15–16). (**C**) ipGTT after 2 weeks of d-serine supplementation (n = 5). (**D**) Random fed blood glucose levels during 15 weeks of d-serine supplementation following 8 weeks on HFD prior to d-serine supplementation (n = 4). At weeks 6 and 12, d-serine supplementation was switched. (**E**) ipGTT after 5 weeks of d-serine supplementation, 6 weeks after the first serine switch and 3 weeks after the second serine switch of the mice shown in (D). (**F**) Insulin tolerance test after 3 weeks of d-serine supplementation (n = 5). (**G**) Random fed serum insulin levels after 3 weeks of d-serine supplementation. (**H**) ipPTT after 5 weeks of d-serine supplementation (n = 5). (**I**) Liver d-serine levels after 8 weeks chronic d-serine supplementation (n = 7–8). Data are shown as mean ± SEM. Statistics were calculated either by t-test, one-way or two-way ANOVA with Tukey's or Sidaks (for Fig 2C and E, F, H) multiple comparison post-hoc test (****P < 0.0001, ***P < 0.001, **P < 0.01, *P < 0.05).

To assess the impact of d-serine on glucose metabolism the different treatment groups were subjected to intraperitoneal glucose tolerance tests (GTT) after six weeks on diet. Both CD and HFD mice supplemented with d-serine were hyperglycemic at baseline and severely glucose intolerant (Figure 2A). Measurement of random fed blood glucose after three and eight weeks revealed elevated glucose levels in mice receiving d-serine (Figure 2B). Glucose tolerance was already impaired after two weeks in mice fed a CD + d-serine (Figure 2C). Similar to the effects of weight gain (Figure 1F), hyperglycemia, and glucose intolerance could be induced in mice with established diet induced obesity and reversed within one week of switching d-serine with water (Figure 2D–E). Intraperitoneal insulin tolerance tests (ITT) did not reveal differences in insulin sensitivity after three weeks of d-serine supplementation in CD fed animals (Figure 2F). Random fed insulin levels were not changed in CD + d-serine animals and reduced to CD levels in HFD + d-serine mice (Figure 2G). Histological analysis of SCF, PGF, and BAT also did not reveal differences in CD + d-serine mice compared to controls, albeit average adipocyte size in PGF and SCF was reduced in HFD + d-serine mice (Figure S2A–B).

d-serine mediated hyperglycemia is independent of insulin sensitivity. Thus, we tested if increased gluconeogenesis, through d-serine metabolism, contributes to hyperglycemia. Pyruvate tolerance tests did not show statistically significant differences in gluconeogenesis in d-serine treated CD fed animals (Figure 2H). Glycogen content was also not different between groups (Figure S2C). NMR based metabolomics of the livers showed increased total serine levels in d-serine treated animals (Figure S2D), reflecting accumulation of d-serine (Figure 2I). Conversely, glycine and the reduced form of glutathione were reduced (Figure S2D). No other statistically significant metabolite differences, including β-hydroxybutyrate, despite a decrease of phosphorylcholine in HFD + d-serine treated animals, were observed (Figure S2E–F). Thus, d-serine alters hepatic one carbon metabolism but does not impair insulin tolerance or hepatic gluconeogenesis.

### 3.3 d-serine impairs insulin secretion from pancreatic β-cells

**Figure 3:**
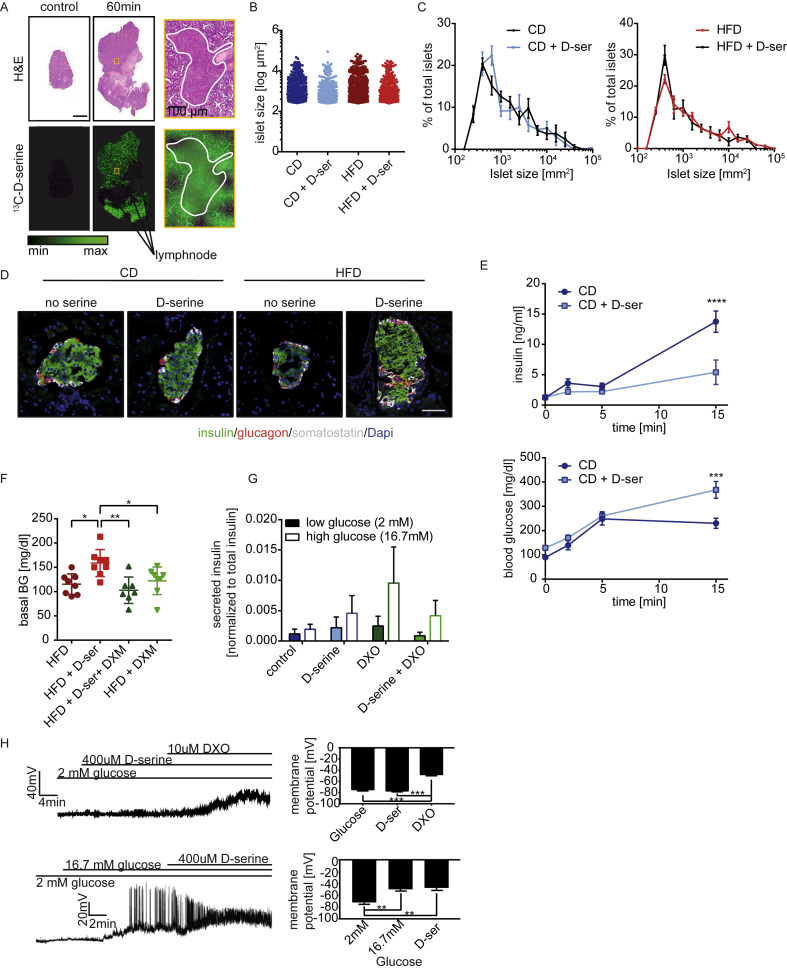
**d****-serine impairs insulin secretion but not islet morphology**. (**A**) 4 week old C57Bl/6 mice were gavaged with 10 mg/kg ^13^C-labeled d-serine and sacrificed after 60 min. Pancreata were prepared for MALDI FT-ICR MSI and stained with H&E. Control animals were gavaged with water. Size bar represents 2 mm. (**B**) Quantification of islet size and (**C**) islet size distribution (n = 4 per group) after 8 weeks of d-serine supplementation. (**D**) Representative triple staining of pancreatic islets after 3 weeks of serine supplementation. White: somatostatin. Red: glucagon. Green: insulin. Blue: Dapi. Size bar represents 50 μm. (**E**) Glucose stimulated insulin secretion after 4 weeks of serine supplementation (n = 5). (**F**) Blood glucose after one week on HFD +/− 10 g/l d-serine and 0.3% dextromethorphan in drinking water following a 4 h fast. (**G**) Glucose stimulated insulin secretion of isolated pancreatic islets pretreated with either 400 μM d-serine, 10 μM Dextrorphan tartrate (DXO) or a combination thereof for 1 h (n = 4). (**H**) Representative current-clamp trace of a mouse pancreatic β-cell displaying the electrical response to 400 μM d-serine and 10 μM DXO, in the presence of 2 or 16.7 mM glucose. The graph on the right shows mean membrane potential (n = 3–5). Values are mean ± SEM, analyzed by one-way or two-way ANOVA with Tukey's or Sidaks (for Fig 3E) multiple comparison post-hoc test (****P < 0.0001, ***P < 0.001, **P < 0.01, *P < 0.05).

To assess the uptake of d-serine into the pancreas, Matrix-assisted laser desorption/ionization Fourier-transform ion cyclotron resonance mass spectrometry imaging (MALDI FT-ICR MSI) of ^13^C-d-serine was used showing uptake into islets of Langerhans and the exocrine pancreas but not peripancreatic lymph nodes (Figure 3A). d-serine supplementation did not affect islet size (Figure 3B), islet size distribution (Figure 3C), or morphology (Figure 3D and Figure S3A). However, d-serine supplementation blunted first and second phase insulin secretion in CD fed animals resulting in hyperglycemia (Figure 3E).

Pancreatic β-cells express NMDA receptors [35], and Marquard and colleagues showed that inhibition of pancreatic NMDA receptors by dextromethorphan (DXM) or its product dextrorphan (DXO) increases insulin secretion *in vitro* and *in vivo* [36]. To test if inhibition of NMDA receptors using DXM can reverse the effects of d-serine on insulin secretion, mice were fed HFD and drinking water supplemented with d-serine, 0.3% DXM or a combination thereof. DXM supplementation prevented d-serine induced hyperglycemia (Figure 3F). However, DXM as well as d-serine and DXM + d-serine treated mice were less glucose tolerant than HFD fed control mice after one week of supplementation (Figure S3B). DXM treated mice had reduced energy expenditure (Figure S3C), but a trend to increased food intake (Figure S3D). Most importantly, however, DXM supplemented mice had significantly lower water intake (Figure S3E) and therefore also reduced d-serine uptake in the co-administrated mice. Thus, the observed normalization of blood glucose could result from reduced d-serine exposure.

To overcome this limitation, the effects of d-serine and NMDA receptor inhibition on GSIS were tested in isolated islets. DXO supplementation increased glucose stimulated insulin secretion, though changes did not reach statistical significance (Figure 3G). In contrast, d-serine or the combination of d-serine and DXO did not alter insulin secretion compared to control islets. Measurements of islet membrane potential corroborated these findings as no changes after addition of d-serine could be observed at low (2 mM) or high (16.7 mM) glucose concentrations (Figure 3H).

### 3.4 d-serine regulates insulin secretion via the sympathetic nervous system

**Figure 4:**
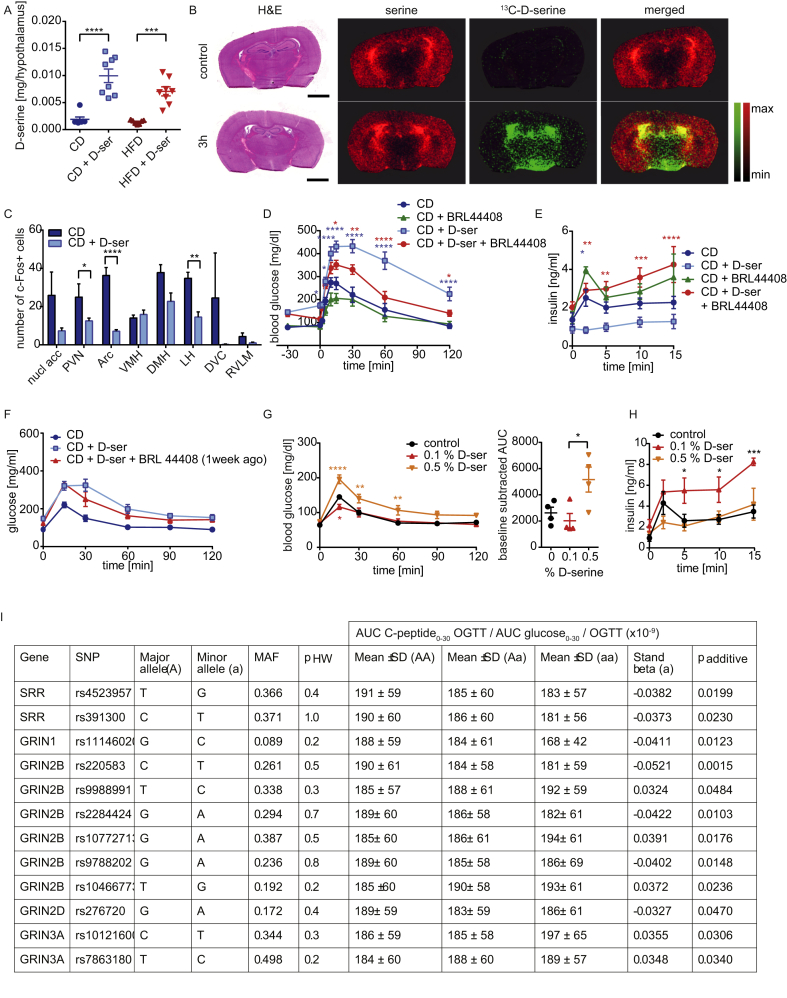
**α2-adrenergic receptor inhibition rescues****d****-serine suppressed insulin secretion**. (**A**) Hypothalamic d-serine levels after 8 weeks of d-serine supplementation. (**B**) 4 week old C57Bl/6 mice were gavaged with 10 mg/kg ^13^C-labeled d-serine and sacrificed after 3 h. Brains were prepared for MALDI FT-ICR MSI and stained with H&E staining. As negative control, animals were gavaged with water. Size bar represents 2 mm. (**C**) c-Fos expression in different brain regions after 6 weeks of d-serine supplementation (n = 1–3). Animals were fasted for 6 h prior to the experiment. (**D**) Glucose and (**E**) insulin levels during a GSIS after 2 and 4 weeks of d-serine supplementation +/− treatment with the α-adrenergic receptor inhibitor BRL 44408 (5 mg/kg) 30 min prior to glucose administration. (n = 5–10). (**F**) ipGTT after 3 weeks of d-serine supplementation and one week after a single injection of BRL 44408 (5 mg/kg) (n = 5). (**G**) ipGTT after 11 days of 0.1 or 0.5% d-serine supplementation (n = 4). (**H**) GSIS after 4 weeks of d-serine supplementation (n = 4). (**I**) Hardy–Weinberg equilibrium of genotype distribution analyzed by χ^2^-test (p _HW_). Genotype-phenotype association was assessed by multiple linear regression analysis (standard least squares method) in the additive inheritance model (p _additive_). Insulin secretion data adjusted for gender, age, BMI, and insulin sensitivity are derived from the linear regression models. The effect size of the minor allele is given as standardized beta. AUC – area under the curve; MAF – minor allele frequency; OGTT – oral glucose tolerance test; SD – standard deviation; SNP – single nucleotide polymorphism. Data are shown as mean ± SEM. Statistics were calculated using either ordinary one-way or two-way ANOVA with Tukey's multiple comparison post-hoc test (****P < 0.0001, ***P < 0.001, **P < 0.01, *P < 0.05).

Thus, d-serine does not appear to regulate insulin secretion primarily via direct action on pancreatic β-cells. Conversely, d-serine action is best described in the CNS. Indeed, we observed significantly elevated d-serine levels in the hypothalamus following eight weeks of d-serine supplementation (Figure 4A) and rapid uptake of ^13^C-d-serine into the hypothalamus following oral gavage (Figure 4B). Chronic d-serine supplementation reduced c-Fos expression in various hypothalamic regions (Figure 4C and Figure S4A). Thus, we tested if d-serine regulates insulin secretion through the sympathetic nervous system. d-serine supplemented animals were injected with the α2 adrenergic receptor inhibitor BRL 44408 [37], [38] prior to assessment of glucose tolerance and insulin secretion. BRL 44408 injection normalized d-serine induced hyperglycemia within 30 min and completely restored glucose tolerance in these mice. BRL 44408 had no significant effects in CD fed animals compared to controls. (Figure 4D). This was accompanied by a substantial increase in insulin secretion (Figure 4E). A GTT performed one week after the single injection of BRL 44408 did not reveal a significant improvement of overall glucose tolerance of BRL 44408 + d-serine mice, but basal glucose levels remained significantly improved (Figure 4F). In contrast to the effects of high dose d-serine supplementation, supplementation with 0.1% d-serine showed the opposite effect resulting in improved glucose tolerance compared to control animals after 11 and 23 days (Figure 4G and Figure S4B), which was due to increased insulin secretion (Figure 4H and Figure S4C).

### 3.5 SNPs in SRR and NDMAR subunits are associated with insulin secretion in humans

To address the relevance of d-serine for insulin secretion in humans, we analyzed the association of 137 common single nucleotide polymorphisms (SNPs) in *SRR*, *DAO*, and the genes encoding Asc-1 (*SLC7A10*), serine hydroxymethyltransferase 1 (*SHMT1*), and NMDAR subunits (*GRIN1*, *GRIN2A*, *GRIN2B*, *GRIN2D*, *GRIN3A*, *GRIN3B*) with insulin secretion in 2,670 prediabetic subjects. After adjusting the linear regression models for gender, age, BMI, and insulin sensitivity, we identified two SNPs in *SRR*, one SNP in *GRIN1*, six SNPs in *GRIN2B*, one SNP in *GRIN2D*, and two SNPs in *GRIN3A* that were nominally associated with insulin secretion estimated from C-peptide levels during an oral glucose tolerance test (Figure 4I).

## 4 Discussion

Here, we show that d-serine regulates glucose homeostasis and weight gain in male C57Bl/6 mice. We demonstrate that orally administrated d-serine rapidly enters the circulation and is taken up by and accumulates in liver, adipose tissue, kidneys, and the hypothalamus. Accumulation of d-serine in adipose tissue, liver, and pancreas did not result in altered tissue morphology or signs of inflammation. However, chronic d-serine treatment altered metabolic regulation of the CNS. We find that d-serine reduces HFD induced weight gain through a reduction in high fat food intake, which seems to depend on altered food preference. These data are in line with Sasaki et al., who described that d-serine suppresses intake of high preference food resulting in blunted weight gain [15]. However, in contrast to this previous study, in our experiments, mice exposed to HFD + d-serine resumed consuming HFD, albeit less than HFD control mice, which could be explained by differences in gut microbiota between and the expansion of obesogenic bacteria facilitating weight gain in our mouse facility.

Most importantly, high-dosed d-serine supplementation resulted in reversible hyperglycemia and glucose intolerance within one to two weeks of supplementation due to impaired insulin secretion. This effect is most likely indirect via activation of the sympathetic nervous system. d-serine supplementation reduced c-Fos expression in several hypothalamic regions. Of special interest, activation of ARC and PVN have previously been shown to increase insulin secretion [39], [40], which is in line with our data showing reduced c-Fos expression and reduced insulin secretion. Moreover, impaired insulin secretion can be corrected by using an α2 adrenergic receptor inhibitor, with a single injection of the inhibitor normalizing basal blood glucose levels for one week. However, d-serine appears to regulate only a subset of sympathetic nerves as we did not observe increased brown adipose tissue function, suggestive for overall increased SNS outflow. In contrast, supplementation with low concentrations of d-serine improved insulin secretion and glucose tolerance. Thus, it is tempting to speculate that chronic exposure to high d-serine concentrations results in some form of d-serine resistance.

Together, we describe the actions of the NMDA receptor co-agonist d-serine in the regulation of weight gain, food intake, and glucose homeostasis. We show that chronic administration of d-serine has beneficial metabolic effects through reduced HFD induced weight gain. Conversely, d-serine reduces glucose stimulated insulin secretion resulting in hyperglycemia and glucose intolerance. These results are of potential importance for schizophrenic patients considering treatment options chronically elevating d-serine levels, as this could pose a significant clinical challenge aggravating the risk for developing type 2 diabetes. Clinical doses of d-serine are ∼20-fold lower than the dose used in this study [41], [42], [43]. However, the negative effects of d-serine on glucose homeostasis most likely require accumulation of d-serine in the CNS. Thus, lower doses could simply take longer to reach the necessary threshold in humans. The same safety concerns also apply for the pharmacological inhibition of DAO, which is in clinical trials to increase d-serine levels [44], [45], [46]. Within this study, we do not provide direct evidence for a role of d-serine or d-serine elevating drugs in the regulation of insulin secretion in humans. However, analysis of SNPs revealed significant associations between SRR, as well as NMDAR subunits, and insulin secretion, strongly suggesting the same or a similar mechanism in humans. In conclusion, we extend the repertoire of metabolically important neurotransmitters by demonstrating that d-serine regulates food intake and glucose homeostasis and provide important safety information for the clinical use of d-serine and potentially a whole class of d-serine elevating antipsychotics.

## Author contributions

LS and SU designed and conducted the experiments and wrote the manuscript. SH and AI performed experiments. AlBr and ME measured d-serine concentrations. TG, BL; CGC and MT performed brain histology and quantification of c-Fos. SSH and PSK performed the NMR metabolomics. EG and TS performed the microbiota analysis. AcBu, AF and AW conducted the MALDI imaging and size distribution analysis of adipocytes and islets. EH and GR performed the electrophysiological analysis on isolated β-cells. HS, MH and HUH performed SNP analysis.

## Funding

This work was supported by iMed the initiative for personalized medicine of the Helmholtz Association and funds from the German Research Foundation as well as from the project Aging and Metabolic Programming (AMPro). This work was supported in part by funding to MHT from the Alexander von Humboldt Foundation, the Helmholtz Alliance *ICEMED* & the Helmholtz Initiative on Personalized Medicine *iMed* by Helmholtz Association, funding by European Research Council ERC (AdG *HypoFlam* no. 695054) and the Helmholtz cross-program topic “Metabolic Dysfunction”. TG received support of the Technische Universität München – Institute for Advanced Study, funded by the German Excellence Initiative and the European Union Seventh Framework Programme under grant agreement no. 291763. GAR was supported by grants from the MRC UK (MR/J0003042/1; MR/N00275X/1; MR/L020149/1 [DIVA]), a Wellcome Trust Senior Investigator (WT098424AIA) and Royal Society Wolfson Research Merit Awards, the Biotechnology and Biological Sciences Research Council (BB/J015873/1) and Diabetes UK Project (BDA11/0004210; BDA/15/0005275) grants.

## Data availability

Data will be uploaded to SRA upon acceptance of the manuscript.
