# Supplementary material for: Chronic d-serine supplementation impairs insulin secretion
Source: Mol Metab. 2018 Jul 25;16:191–202. doi: 10.1016/j.molmet.2018.07.002 (PMC6157639; doi:10.1016/j.molmet.2018.07.002)

Supplemental Figure 1 related to Figure 1

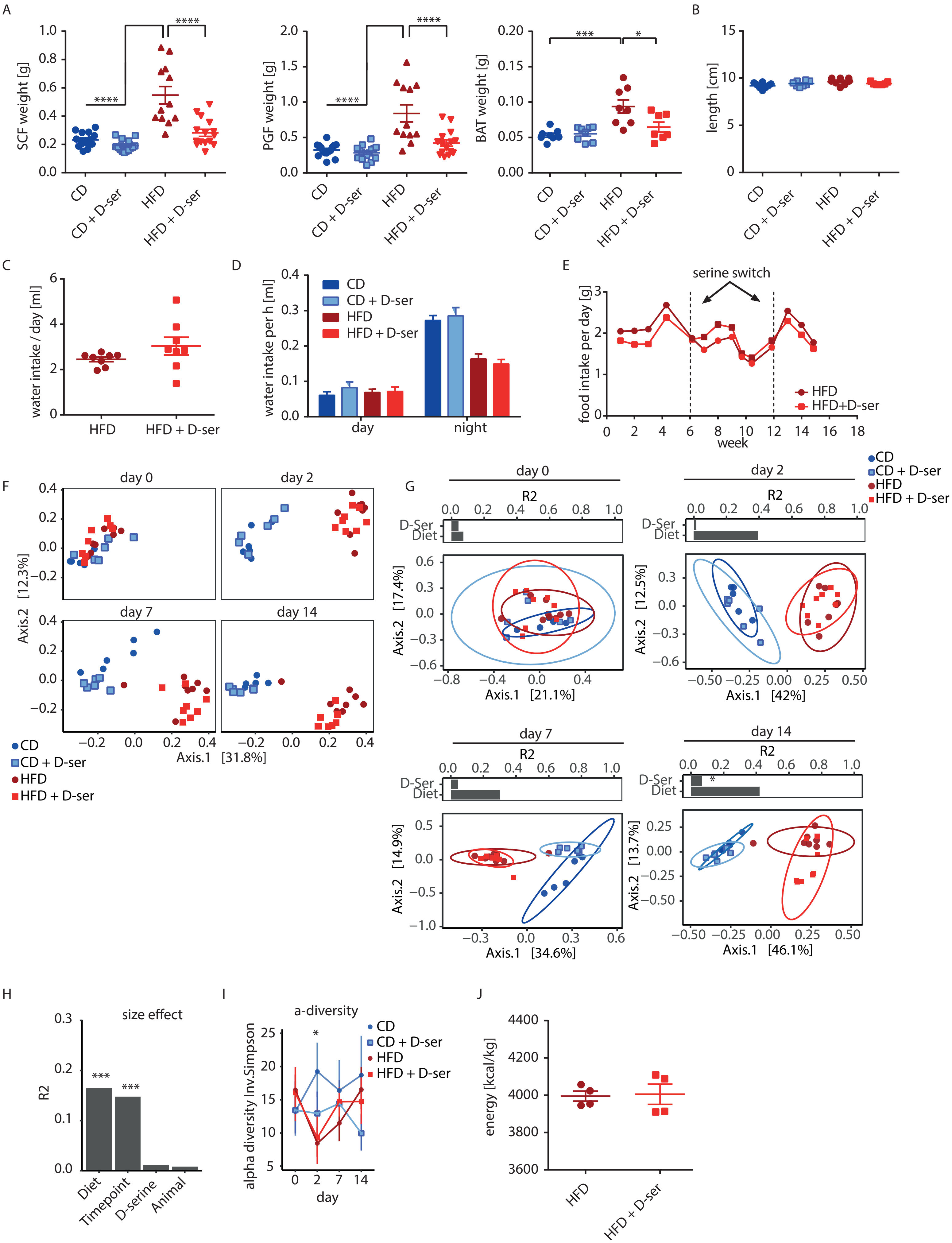

Supplemental Figure 2 related to Figure 2

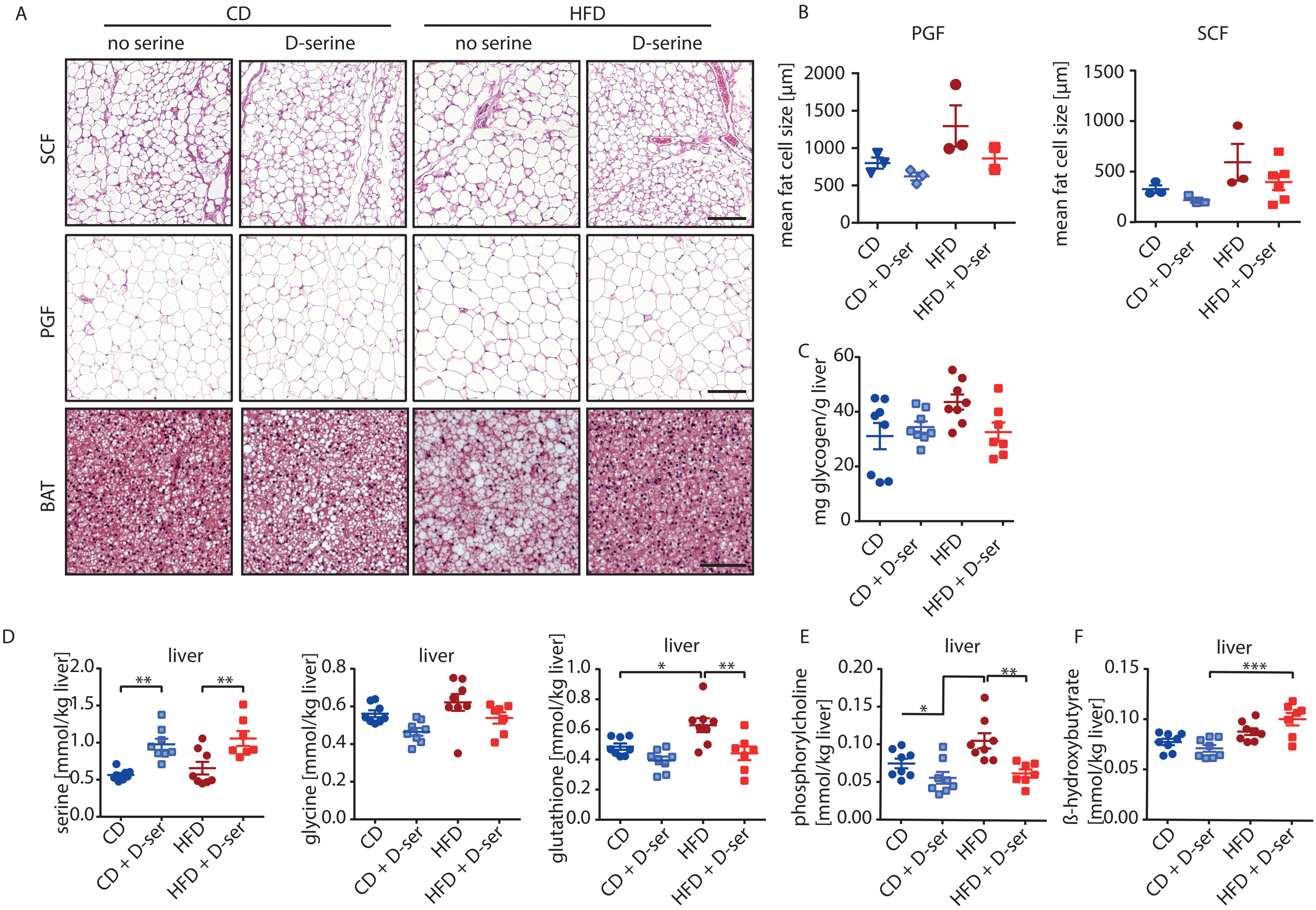

Supplemental Figure 3 related to Figure 3

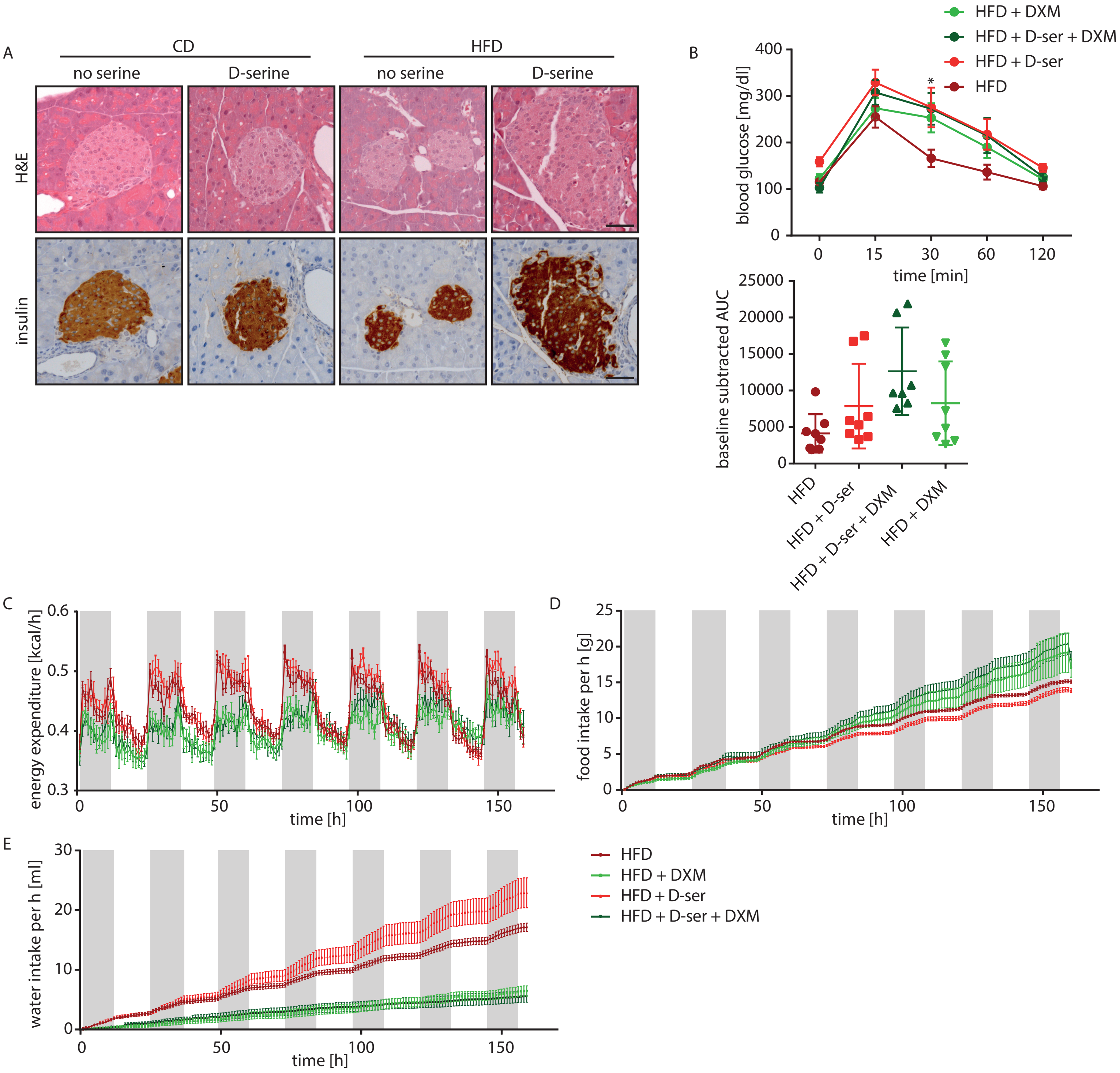

Supplemental Figure 4 related to Figure 4

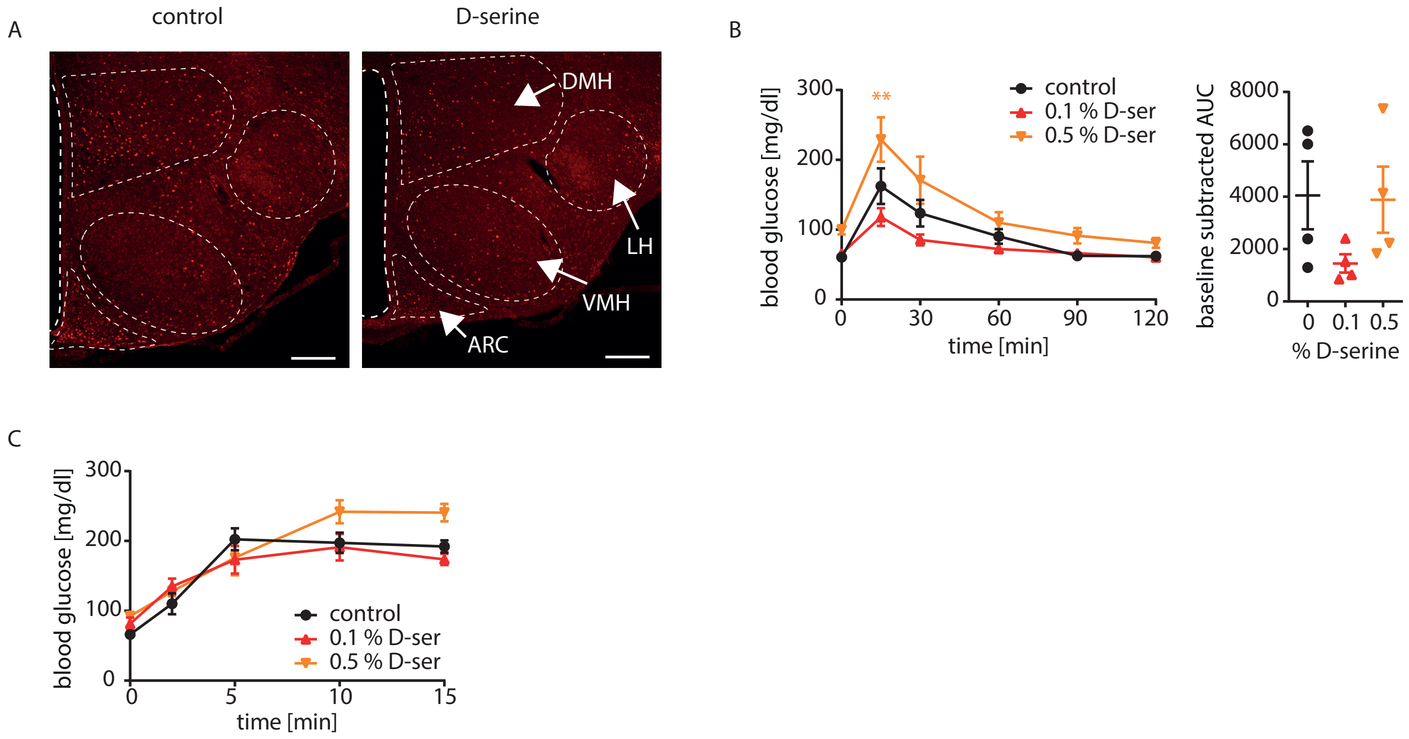

Supplement: Multimedia component 1 [file mmc1.pdf]
